# Impact of Infrabony Defects Treatment on Periodontal Markers and Glycated Hemoglobin Using Platelet-Rich Fibrin, Guided Tissue Regeneration, and Access-Flap Surgery

**DOI:** 10.3390/medicina60111769

**Published:** 2024-10-29

**Authors:** Ada Stefanescu, Irina-Georgeta Sufaru, Cristian Martu, Diana-Maria Anton, Gabriel Rotundu, Kamel Earar

**Affiliations:** 1 Faculty of Dental Medicine, “Dunarea de Jos” University, Al. I. Cuza Street 35, 800216 Galati, Romania; 2 Faculty of Dental Medicine, “Grigore T. Popa” University of Medicine and Pharmacy, Universitatii Street 16, 700115 Iasi, Romania; 3 Faculty of Medicine, “Grigore T. Popa” University of Medicine and Pharmacy, Universitatii Street 16, 700115 Iasi, Romania; 4 “St. Andrew” Emergency Clinical County Hospital, Brailei Street 177, 800578 Galati, Romania

**Keywords:** access flap, infrabony defects, periodontal clinical parameters, periodontitis, platelet-rich fibrin, type II diabetes, wound healing

## Abstract

*Background and Objectives*: This study evaluated the outcomes of single open-flap debridement, open-flap debridement (OFD) plus resorbable membrane placement, and OFD with resorbable membrane placement plus platelet-rich fibrin (PRF) in terms of periodontal clinical parameters and glycated hemoglobin (HbA1c) levels in 24 adult patients with stage 3 grade C periodontitis and type II diabetes mellitus. *Materials and Methods*: The primary outcome measure for this study was the clinical attachment level (CAL); secondary outcomes included additional periodontal parameters, such as the plaque index (PI), bleeding on probing (BOP), probing depth (PD), as well as glycated hemoglobin (HbA1c) levels to evaluate the systemic impact of the treatments on glycemic control. The parameters were assessed before and at three and six months post-surgery. In Group A, the flap was sutured closed; in Group B, an absorbable collagen membrane was placed over the defect; and in Group C, PRF was utilized in the defect, with two additional PRF membranes used to cover the defect. The wound healing index (WHI) was recorded at 7 and 14 days after the surgery. *Results*: The initial findings indicated no significant differences in the periodontal parameters among the three groups. However, improvements in the PD and CAL were most notable in Group C, followed by Group B, with Group A showing the slightest improvement. At six months, there was a highly significant difference in the CAL (*p* < 0.001). Group C (4.92 ± 0.35) and Group B (4.99 ± 0.31) demonstrated the most significant improvements in the CAL compared to Group A (5.89 ± 0.57). At seven days post-surgery, Group C demonstrated significant healing, with 85% of the sites showing complete healing. By the 14-day mark, all sites in Group C indicated complete healing. Although the HbA1c values did not exhibit statistically significant differences among the groups at baseline, at the 6-month evaluation, all groups showed significantly lower values than baseline. However, the comparison between groups revealed significantly improved values for Group C. *Conclusions*: The study’s results suggest that PRF is an exceptional material for infrabony defects treatment and notably improves HbA1c levels.

## 1. Introduction

Diabetes has been proven to be a significant risk factor for periodontitis [1]. A two-way relationship exists between periodontal disease and diabetes, and appropriate periodontal care can positively impact the metabolic status of diabetic patients [1]. While the bidirectional relationship between periodontitis and diabetes has been extensively studied, it is essential to note that patients with periodontitis, even in the absence of diabetes, tend to have higher levels of HbA1c compared to individuals without periodontitis and without diabetes [2]. This finding underscores the broader impact of periodontal disease on metabolic health and further supports the rationale for monitoring glycemic levels in patients with periodontitis, regardless of their diabetic status. Multiple meta-analyses consistently demonstrate that effective periodontal therapy can reduce glycated hemoglobin (HbA1c) levels. Notably, a meta-analysis from 2010, which evaluated five individual studies involving 371 patients, revealed a significant mean decrease in HbA1c levels of 0.40% [3].

This significant reduction in HbA1c was observed over a 3- to 9-month follow-up after periodontal therapy, as reported by Teeuw et al. [3]. This finding suggests that appropriate periodontal intervention can improve oral health and positively influence blood glucose control in patients with diabetes.

A population-based study involving a cohort of over 5000 individuals with diabetes revealed that those who underwent at least one session of access periodontal surgery exhibited a 0.25% reduction in HbA1c levels compared to their counterparts who did not undergo the surgical procedure [4]. These observations are highly relevant, as reducing HbA1c levels is associated with a lower risk of diabetes-related complications [5]. Studies show that each 1% reduction in HbA1c is associated with a significant decrease in the risk of diabetes-related complications, including diabetes-related deaths, myocardial infarction, and microvascular complications [6].

These findings suggest that periodontal therapy may play a significant role in managing diabetes and improving glycemic control. Therapeutic approaches targeting the health of periodontal tissue could positively impact the overall health of patients with diabetes, thus helping to reduce the risk of complications associated with this condition.

With technological advancements and the modernization of dentistry, patient demands have increased over time. Health awareness of every individual has increased. Added to this is a demand for comfort and aesthetics of the therapeutic solution.

Over the years, periodontics has adapted to the growing demand for improved healing, more comfortable treatment options, management of marginal tissue recessions, and enhanced aesthetic outcomes. This has become one of the main goals of periodontology. Periodontal regeneration is a complex process involving various technologies and materials [7,8,9]. Autogenous bone graft and platelet concentrates are two of the most frequently used techniques that have proven effective in restoring the function of lost tissue and improving the prognosis of the individual tooth [10].

While the overall benefits of periodontal treatment on systemic health are widely accepted, there remains a critical gap in the literature regarding the comparative efficacy of different therapeutic approaches for specific periodontal defects in diabetic patients. Infrabony defects, a common manifestation of advanced periodontitis, present unique challenges regarding treatment success [11]. Few studies have directly compared the outcomes of various treatment modalities for infrabony defects in diabetic populations [12,13].

This study aims to address this gap by comparing the outcomes of three treatment modalities—open-flap debridement (OFD), OFD plus resorbable membrane placement, and OFD with resorbable membrane placement plus platelet-rich fibrin (PRF). Focusing on intrabony defects, which are challenging to treat due to vertical bone loss, and including regenerative materials such as PRF brings a novel perspective to the current understanding of periodontal therapy in diabetic patients. By evaluating the clinical and systemic effects of these therapies, this study contributes valuable new insights into the most effective treatment strategies for periodontal regeneration in patients with type II diabetes.

Platelet-rich fibrin (PRF) is an autologous platelet concentrate similar to a scar matrix that guides the various cellular elements and releases growth factors and cytokines near the injury [14]. This technique, advocated by specific authors, remains a little-studied topic and raises many questions the present study tried to answer.

Thus, the present study proposed to investigate the effects of infrabony defect therapy in patients who underwent only OFD, OFD plus resorbable membrane placement, and OFD plus PRF on periodontal clinical parameters and glycated hemoglobin (HbA1c) in patients with periodontitis and type II diabetes. The null hypothesis of the study states that there are no statistically significant differences in the clinical attachment level (CAL), plaque index (PI), bleeding on probing (BOP), probing depth (PD), wound healing index (WHI), or HbA1c levels between the three treatment groups (OFD, OFD plus resorbable membrane placement, and OFD plus resorbable membrane placement with PRF) at any time point (baseline, three months, and six months post-surgery).

## 2. Materials and Methods

### 2.1. Study Population

This study was a prospective, randomized, controlled, and blinded clinical trial. Twenty-four adult patients with type II diabetes with stage 3, grade C periodontitis who underwent phase I periodontal therapy were enrolled in this study based on the following inclusion criteria: a good level of oral hygiene (plaque index < 1); three different molars in three different quadrants of the oral cavity, in the same patient, with a 2-walled or 3-walled infrabony defect at a probing depth (PD) ≥ 6 mm and ≥3 mm; no teeth with furcation lesions or having a degree of mobility greater than 3 mm. This study specifically enrolled patients with infrabony defects, a common manifestation of advanced periodontitis characterized by vertical alveolar bone loss. These defects were selected due to their need for regenerative therapy.

One of the key criteria, including molars in three different quadrants, was chosen to standardize the distribution of the periodontal defects across the study population. This criterion was implemented to reduce variability in the treatment responses arising from differences in tooth location and load distribution across the oral cavity.

Molars are particularly susceptible to infrabony defects due to their anatomical complexity and higher functional load. By ensuring that each patient had molars with defects in multiple quadrants, we aimed to balance the severity of periodontal disease across the groups, thus reducing the confounding variables related to tooth position or load-specific responses. This approach helps to isolate the effects of the periodontal treatments being compared, providing a more robust comparison of their efficacy in managing infrabony defects.

The following exclusion criteria were applied: patients who had contraindications to surgery, patients who smoke, pregnant patients or nursing mothers, patients with immunosuppression or systemic diseases other than type II diabetes, use of medications known to affect periodontal or systemic health (e.g., immunosuppressants, anticoagulants), and patients with drug allergies. These criteria were established to ensure a more homogeneous sample and to limit biases that could influence the clinical and systemic outcomes.

The diagnosis of diabetes was confirmed by the diabetologist, who performed a rigorous assessment of the patient’s health.

This study was designed and conducted according to the ethical principles and international standards in the field of medical research. All study participants were fully informed about the aims, risks, and benefits involved in the research, and their written consent was obtained before their inclusion in the study. The data for this study were collected in the clinical sections of the dental faculties in Iasi and Galati, where all periodontal assessments, treatments, and follow-up evaluations were conducted.

This study fully complied with the principles of the Declaration of Helsinki of 1975, revised in 2013, having the Approval of “Grigore T. Popa” University Research Ethics Commission CEC/30.07.2020.

### 2.2. Evaluation of Clinical Periodontal Parameters

The clinical periodontal parameters, including the plaque index (PI), gingival bleeding on probing (BOP), probing depth (PD), and clinical attachment level (CAL), were assessed before surgery (T0) and then at three months (T1) and six months (T2) after the procedure by a specialist who was unaware of the treatment performed.

The plaque index (PI) was evaluated according to the criteria established by Löe and Silness [15] to assess the degree of plaque accumulation on the tooth surface. The PD and CAL were measured using a UNC 15 periodontal probe (Henry Schein, Gillingham, Kent, UK), providing a detailed assessment of the patient’s periodontal status. The PD was determined as the distance from the gingival margin to the base of the periodontal pocket. At the same time, the CAL was recorded as the distance from the enamel-cementum junction (CEJ) to the base of the periodontal pocket, reflecting periodontal attachment loss. BOP was recorded as the presence of bleeding during periodontal probing within 30 s after the PD measurement [16].

A single examiner (I.-G.S.) clinically assessed all these parameters to ensure consistency and accuracy of the measurements. To minimize variations and ensure adequate reproducibility, the examiner underwent a calibration procedure before the start of the study. Intra-examiner calibration was performed on a subset of 10% of patients, consisting of duplicate measurements of the PD and CAL 48 h apart. An examiner was considered calibrated when a statistically significant correlation and a statistically insignificant difference were observed between duplicate measurements (r = 0.85 for PD and 0.90 for CAL), thus indicating the consistency and reliability of the measurements performed.

### 2.3. HbA1c Analysis

This study assessed the glycated hemoglobin A1c (HbA1c) level for each patient before surgery and at three and six months post-surgery. In this study, HbA1c levels were measured using the immunoturbidimetry method. This method was selected due to its reliability and widespread use in clinical settings for assessing long-term glycemic control. While other methods, such as high-performance liquid chromatography (HPLC), are available, immunoturbidimetry was chosen for its cost-effectiveness, ease of use, and established validation in diabetes care [17]. This method ensures the consistency and comparability of HbA1c measurements across the study population.

### 2.4. Surgical Procedures

An experienced operator (A.S.) performed the surgical procedure. After administering local anesthesia, intra-crevicular incisions were made, extending to the adjacent teeth, with particular attention paid to the maximum preservation of the interdental gingival tissue. The surgical procedures in this study were performed using incisions according to the modified papilla preservation flap (MPPF) technique, as described by Cortellini et al. [18]. This technique was chosen to preserve the interdental papilla during flap surgery, optimizing the regenerative potential and aesthetic outcomes in treating intrabony defects. The full-thickness buccal and oral flaps were trimmed, and care was taken to remove all granulation tissue from the defect without interfering with the bone contour. The roots were planed using hand instruments. This procedure was standard for each of the three teeth from each patient included in the study.

In Group A (control group, *n* = 24 sites), after completing the described operation, the flap was sutured in the initial position, completely closed with vertical or horizontal sutures with 5.0 thread (Dafilon 5.0. monofilament and uncoated polyamide, B. Braun Surgical S.A. Barcelona, Spain). Thus, each patient presented a tooth with an infrabony defect treated only with an OFD.

In Group B (test group, *n* = 24 sites), before suturing the flap, an absorbable collagen membrane (T-Gen, SK Bio Land Co. Ltd., Cheonan, Republic of Korea) was positioned over the infrabony defect, conformed to fit, and secured to the defect using absorbable polyglycolic acid thread (D-Tek 5.0, Demophorius, Limassol, Cyprus).

In Group C (test group, *n* = 24 sites), platelet-rich fibrin (PRF) of the necessary dimensions was inserted into the infrabony defect, and two additional PRF membranes were employed to envelop the defect, thereby stimulating the interface between the gingival tissue and the root surface across the entire height of the flap.

The randomization process was conducted using a computer-generated randomization sequence to assign patients into one of the three groups: (1) OFD alone (control), (2) OFD plus resorbable membrane placement, or (3) OFD with resorbable membrane placement plus platelet-rich fibrin (PRF). To ensure allocation concealment, sealed, opaque envelopes containing the treatment group assignments were prepared in advance and opened only at the time of surgery by the clinician performing the procedure. Regarding masking, the clinical evaluators who assessed the periodontal parameters (plaque index, bleeding on probing, probing depth, clinical attachment loss) and glycated hemoglobin (HbA1c) levels were blinded to the treatment group allocations throughout the study to minimize bias.

### 2.5. Postoperative Care

All patients received antibiotic treatment for one week, administered twice daily, according to the standard postoperative care protocol. The antibiotic used was Augmentin Duo, which contains 875 mg of amoxicillin and 125 mg of clavulanic acid (GlaxoSmithKline, UK).

Postoperative care used a 0.2% chlorhexidine solution (Curasept ADS 220, Curaden AG, Kriens, Switzerland) rinsed twice daily for two weeks. This aspect is essential for maintaining proper oral hygiene and reducing the risk of infection immediately following surgery.

According to the standard postoperative care schedule, the sutures were removed 14 days after the intervention. The participants also received clear and detailed instructions regarding maintaining adequate oral hygiene in the postoperative period, which has been reinforced to ensure proper understanding and compliance by the patients.

To monitor progress and evaluate the surgery long-term, participants were scheduled for weekly follow-up visits for one month after surgery and at regular intervals of three and six months after that.

### 2.6. Assessment of Wound Healing

The wound healing index (WHI) was recorded by the same investigator who examined the periodontal status of the selected teeth (I.-G.S.) at 7 and 14 days after surgery, closely following the progress of the healing process. This index assessed critical aspects of the wound healing process, providing detailed insight into postoperative progress.

To ensure the consistency and objectivity of the evaluation, the investigator awarded a score from 1 to 3, according to the established criteria [19]:

- A score of 1 was assigned for excellent healing, characterized by the absence of gingival edema, erythema, suppuration, patient discomfort, or flap dehiscence. This category indicates an ideal healing process without significant complications.
- A score of 2 was given for patchy or partial healing, with mild symptoms such as gingival edema, erythema, discomfort, or flap dehiscence but without suppuration. This category reflects ongoing healing with significant progress but requires additional monitoring and care.
- A score of 3 was reserved for cases of evident poor healing, where significant gingival edema, erythema, patient discomfort, flap dehiscence, and suppuration were present. This category indicates a suboptimal healing process, which may require additional interventions or changes in the treatment protocol.

### 2.7. Sample Size Calculation

The sample size calculation was performed to ensure sufficient statistical power for detecting significant differences in the primary outcome, the CAL. Based on the study by Gamal et al. [20], we anticipated a mean difference of 1.5 mm in CAL improvement between treatment groups, with an estimated standard deviation of 1.0 mm. Using an alpha significance level of 0.05 and a power of 0.85, it was determined that a minimum of 20 sites per treatment group would be required to detect significant differences. To account for potential subject loss throughout the study or dropouts during follow-up, we included an additional 20% in the sample size calculation. This adjustment increased the total sample size to 24 sites per group, ensuring sufficient accuracy and compensating for possible dropouts.

### 2.8. Statistical Analysis

The primary outcome measure for this study was the CAL; secondary outcomes included additional periodontal parameters, such as the PI, BOP, PD, and WHI, as well as HbA1c levels to evaluate the systemic impact of the treatments on glycemic control in patients with type II diabetes.

To evaluate the effectiveness of the treatment and to analyze the changes in periodontal parameters and HbA1c level, we used descriptive statistics and analysis of variance with repeated measures ANOVA. We used paired *t*-tests to examine the changes within the same group over time. We also applied the Chi-square test to evaluate the differences in the wound healing index between the surgically treated groups at 7 and 14 days after surgery. The statistical significance level was set below 0.05 for all tests performed.

**Figure 1:**
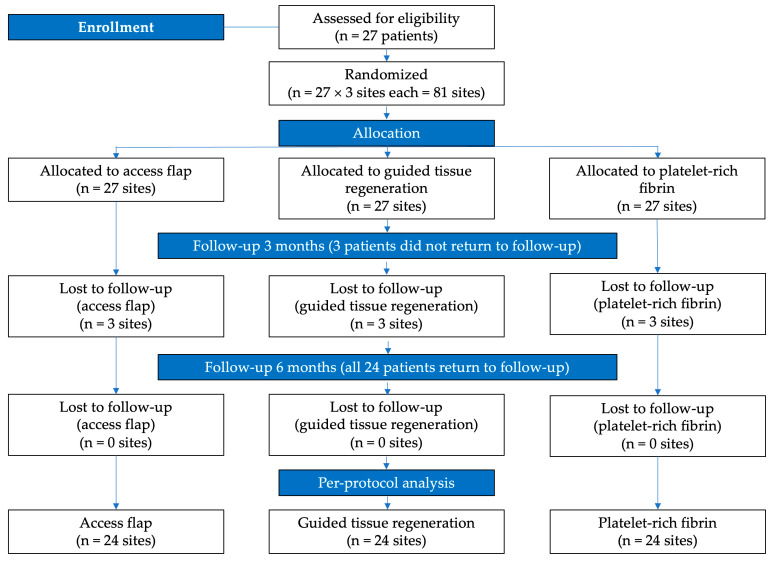
Study’s flowchart.

The study’s flowchart is depicted in Figure 1.

## 3. Results

### 3.1. Demographic Results

We initially screened 27 patients eligible for inclusion in the study. Three patients did not attend postoperative visits, so the statistical analysis was performed on a final number of 24 patients.

The gender in the study group was 13 male patients (54.17%) and 11 female patients (45.83%), with a mean age of 45 ± 1.35 years. Among them, 17 came from the urban environment (70.83%) and seven from the rural environment (29.17%) (Table 1).

### 3.2. Clinical Results

#### 3.2.1. Plaque Index (PI)

At baseline (T0), the PI values were not significantly different between the groups (Group A: 0.82 ± 0.23, Group B: 0.78 ± 0.19, Group C: 0.77 ± 0.17, *p* = 0.532). All groups had comparable plaque levels at the study’s beginning (Table 2).

At three months (T1), there was a slight reduction in the PI across all groups (Group A: 0.73 ± 0.27, Group B: 0.69 ± 0.18, Group C: 0.68 ± 0.19), but the differences were still not statistically significant (*p* = 0.579) (Table 2). Group A’s mean difference was 0.08 ± 0.20 (*p* = 0.221), showing a small statistically insignificant reduction. In Group B, the mean difference was 0.08 ± 0.21 (*p* = 0.041), indicating a small but statistically significant reduction. The mean difference in Group C was 0.08 ± 0.24 (*p* = 0.055), nearing significance but not quite reaching statistical significance (Table 3).

At six months (T2), a significant difference was observed (*p* = 0.028), with Group B (0.53 ± 0.27) and Group C (0.55 ± 0.30) showing more significant reductions compared to Group A (0.70 ± 0.21) (Table 2). The mean difference in Group A was 0.09 ± 0.15 (*p* = 0.001), a statistically significant improvement over the baseline. Group B showed a mean difference of 0.23 ± 0.18 (*p* < 0.001), significantly reducing the PI. The mean difference in Group C was 0.19 ± 0.17 (*p* < 0.001), also showing a significant improvement, highlighting the role of PRF in plaque reduction (Table 3).

#### 3.2.2. Bleeding on Probing (BOP)

At baseline (T0), the BOP values were similar across all groups (Group A: 4.49 ± 0.61, Group B: 4.42 ± 0.65, Group C: 4.49 ± 0.71, *p* = 0.814), indicating no initial differences (Table 2).

At three months (T1), there was a notable reduction in BOP in all groups, although the differences were not statistically significant (Group A: 3.29 ± 0.87, Group B: 3.02 ± 0.95, Group C: 2.95 ± 0.78, *p* = 0.363) (Table 2). The mean difference in Group A was 1.18 ± 0.97 (*p* < 0.001), indicating a significant reduction in gingival bleeding. In Group B, the mean difference was 1.38 ± 0.87 (*p* < 0.001), showing a slightly greater reduction compared to Group A. Group C showed a mean difference of 1.51 ± 0.64 (*p* < 0.001), indicating the most significant improvement in BOP (Table 3).

At six months (T2), a significant difference emerged between the groups (*p* = 0.001). Groups B (2.32 ± 0.69) and C (2.32 ± 0.58) showed greater reductions in BOP compared to Group A (2.95 ± 0.79), suggesting that the resorbable membrane and PRF contributed to better control of gingival bleeding and inflammation (Table 2). In Group A, the mean difference was 1.51 ± 1.03 (*p* < 0.001), a further significant reduction in BOP compared to the baseline. The mean difference in Group B was 2.07 ± 0.81 (*p* < 0.001), showing a greater reduction compared to Group A. Group C showed a mean difference of 2.14 ± 0.75 (*p* < 0.001) (Table 3).

#### 3.2.3. Probing Depth (PD)

At baseline (T0), the PD values were comparable across the groups (Group A: 7.42 ± 0.63, Group B: 7.62 ± 0.57, Group C: 7.59 ± 0.47, *p* = 0.552) (Table 2).

At three months (T1), the PD was statistically significantly reduced (*p* = 0.019). Group B (5.32 ± 0.71) and Group C (5.22 ± 0.38) showed greater reductions compared to Group A (5.54 ± 0.53) (Table 2). Group A showed a mean difference of 1.85 ± 0.56 (*p* < 0.001), indicating a significant reduction in probing depth. The mean difference in Group B was 2.28 ± 0.51 (*p* < 0.001). Group C presented a mean difference of 2.35 ± 0.61 (*p* < 0.001) (Table 3).

At six months (T2), the differences became more pronounced (*p* = 0.005), with Group C (4.29 ± 0.62) and Group B (4.39 ± 0.55) showing significantly greater reductions in the PD compared to Group A (4.85 ± 0.81) (Table 2). The mean difference for Group A was 2.55 ± 1.21 (*p* < 0.001), while Groups B and C showed mean differences of 3.21 ± 0.76 (*p* < 0.001) and 3.28 ± 0.77 (*p* < 0.001), respectively (Table 3).

#### 3.2.4. Clinical Attachment Level (CAL)

At baseline (T0), the CAL values were not significantly different between the groups (Group A: 8.12 ± 0.83, Group B: 8.19 ± 0.24, Group C: 8.25 ± 0.37, *p* = 0.831), indicating no initial differences (Table 2).

At three months (T1), a trend toward improvement was observed, though not yet statistically significant (*p* = 0.063). Group C (6.02 ± 0.73) showed better CAL outcomes compared to Group A (6.52 ± 0.46) and Group B (6.19 ± 0.56) (Table 2). The mean differences in Groups A, B, and C were 1.58 ± 0.79 (*p* < 0.001), 1.97 ± 0.87 (*p* < 0.001), and 2.21 ± 0.77 (*p* < 0.001), respectively (Table 3).

At six months (T2), there was a highly significant difference in the CAL (*p* < 0.001). Group C (4.92 ± 0.35) and Group B (4.99 ± 0.31) demonstrated the greatest improvements in CAL compared to Group A (5.89 ± 0.57) (Table 2). Groups A, B, and C showed mean differences of 2.21 ± 1.17 (*p* < 0.001), 3.18 ± 0.70 (*p* < 0.001), and 3.31 ± 0.59 (*p* < 0.001), respectively (Table 3).

#### 3.2.5. The Wound Healing Index (WHI)

**Figure 2:**
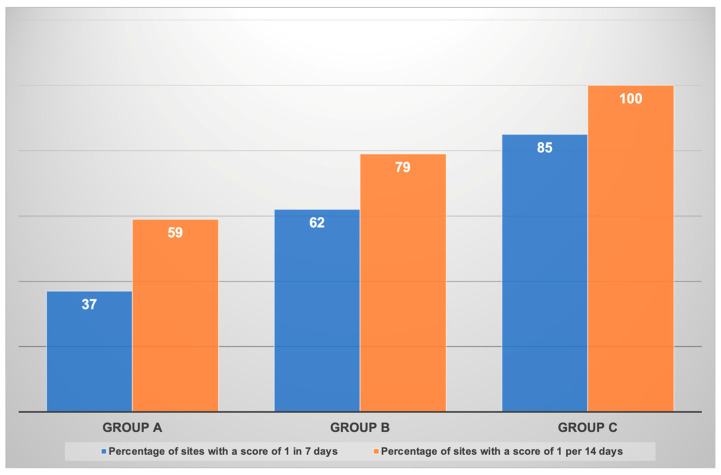
Percentage distribution of healing index with a score of 1 at 7 and 14 days postoperatively.

The wound healing index (WHI) at 7 and 14 days postoperatively is shown in Figure 2. At seven days, Group C showed significant healing, with 85% of the sites showing complete healing, scored as 1. These percentages were 62% and 37% for Groups B and A, respectively (*p* < 0.001).

At the end of 14 days post-intervention, all sites (100%) in Group C showed complete healing with a WHI score of 1, followed by Group B (79%) and Group A (59%). These differences were statistically significant (*p* < 0.001). There were no complications involving the treated teeth at either 7 or 14 days (Figure 2).

### 3.3. HbA1c Results

The HbA1c values did not show statistically significant differences between the groups at the baseline (Table 4). However, at the 3-month assessment (T1), we noted significant differences between the three groups, with significantly lower values for Group C.

At the 6-month assessment (T2), all groups showed significantly lower values than the baseline, but a comparison between groups revealed significantly better values for Group C.

## 4. Discussion

This study was conducted to investigate the impact of therapy for infrabony defects in patients with periodontitis and type II diabetes. The primary objective of this study was to assess the effects of three treatment approaches: OFD alone, OFD accompanied by resorbable membrane placement, and OFD accompanied by PRF on the periodontal clinical parameters and the level of glycated hemoglobin (HbA1c) in the patients. This research was significant not only in the context of periodontitis and type II diabetes but also in understanding how combination therapies can impact clinical outcomes and biochemical parameters in a specific patient population.

In this study, PRF was selected as the platelet concentrate for treating infrabony defects. PRF was chosen due to its favorable balance between ease of preparation, cost-effectiveness, and regenerative potential. Unlike other platelet concentrates, such as advanced platelet-rich fibrin (A-PRF) or leukocyte- and platelet-rich fibrin (L-PRF), PRF can be prepared with minimal manipulation and without the need for anticoagulants or additional additives. This results in a fibrin matrix rich in growth factors, which can enhance wound healing and tissue regeneration.

While A-PRF and L-PRF offer certain benefits, such as an increased leukocyte content or an extended release of growth factors, PRF was chosen for its simplicity, cost-efficiency, and proven efficacy in periodontal regeneration [7]. The decision to use PRF aligns with the aim of this study to explore practical and accessible treatment options that can be readily implemented in clinical practice.

Data from 24 patients with type II diabetes, comprising 72 selected teeth, were analyzed. Each patient was randomized to one of three different surgical approaches applied to three molars chosen from three distinct jaw quadrants. This method was adopted to control inter-patient variability in the three bone defects in the same treated subject.

We chose three molars from different jaw areas for each patient to ensure the treatment was representative and evaluated its effectiveness in various parts of the mouth. We randomly assigned the molars to different surgical approaches to avoid bias or subjective preference in treatment allocation.

At the outset of the study, all clinical and serological parameters indicated no significant differences among the three groups, establishing a fair basis for comparing the effects of the three surgical approaches. This initial consistency facilitated a more confident investigation of the impact of different treatments on patients and specific periodontal and serological parameters.

The results of this clinical trial suggest that the three treatment groups, treated with the PRF + access flap, GTR, or a single-access flap, demonstrated significant improvement in both clinical parameters and HbA1c from the baseline to 6 months and favorable recovery up to 14 days postoperatively. Regarding the inter-group comparisons, the PRF + access flap group exhibited the most significant improvements in both clinical parameters and HbA1c, as well as highly favorable healing, followed by the GTR group. The access flap-only group showed the most minor improvement.

Patients were provided with comprehensive oral hygiene instructions at each study visit. These instructions included recommendations and focused on ensuring that the patients comprehended and correctly applied them at home throughout the study period. This proactive and consistent approach encouraged the patients to maintain proper oral hygiene and regularly monitor their oral condition, resulting in positive outcomes across all three treatment groups. The significant improvements in indices such as the plaque index (PI) and bleeding on probing (BOP) in all three treatment groups highlight the effectiveness of the implemented oral hygiene strategies and indicate the patients’ commitment to compliance. The reduction in these clinical indicators suggests an enhanced oral health status and a positive evolution in managing periodontal disease among the patients involved.

All three treatment groups displayed positive postoperative healing, with no adverse complications noted in any of the cases. Our study results are in line with those of Patel et al. [21], who reported similar findings regarding healing and the absence of complications. Patel et al. [21] observed that 100% of the tooth sites treated with PRF had WHI (wound healing index) scores of 1 at 7 and 14 days postoperatively, indicating favorable healing and no signs of inflammation or infection at the operative site. In contrast, in the single-access flap group, only 38% and 70% of the sites achieved similar scores at the same time intervals.

Sharma and Pradeep presented evidence of the efficacy of platelet-rich fibrin (PRF) in promoting the healing of periodontal lesions [22]. The observed enhancements in the wound healing process are attributed to the elevated concentration of growth factors and dense fibrin present in PRF. This leads to increased stability and rapid neoangiogenesis, promoting faster and more complete recovery of damaged tissue. It is worth noting that the improvements observed in Patel and Sharma’s studies were significantly greater than those observed in our study at six months. These differences may be attributed to variations in the inclusion criteria.

The reported clinical outcomes of the access flap show significant variations across the studies highlighted in the literature. This variation is mainly attributable to differences in the surgical technique and individual factors specific to the patients and surgeons involved. Our study observed that using the GTR technique with a collagen membrane led to improved clinical parameters compared to using the access flap alone to treat periodontal bone defects.

The findings are in line with a systematic review conducted by Stoecklin-Wasmer et al. [23], which showed that GTR with a collagen membrane resulted in significant improvements in clinical outcomes compared to using a single-access flap. It is important to note, however, that in our study, the difference in reduction in PD and CAL between GTR with a resorbable membrane and the use of an access flap was less than what was reported by Stoecklin-Wasmer on healthy individuals, being below 1.52 mm and 1.58 mm, respectively. This observation may be attributable to variations in patient populations, specifically between diabetic and non-diabetic individuals.

These findings emphasize the potential advantages of using the guided tissue regeneration technique with a collagen membrane to address periodontal bone defects. However, it is important to note that the results may differ based on individual patient characteristics, surgeon skill, and other procedural variables. Further research is necessary to identify the most efficient and personalized treatment approaches for patients with these periodontal conditions.

In the study by Ustaoglu et al. [24], it was reported that in the PRF group, reductions in probing depth (PD) and gingival attachment loss (CAL) were 4.69 ± 1.34 mm and 4.19 ± 1.05 mm, respectively. In the GTR group, these reductions were 5.67 ± 1.21 mm and 5.50 ± 1.53 mm, respectively, while in the group treated only with the access flap, the reductions were 3.36 ± 1.12 mm and 3.30 ± 1.17 mm, respectively. These results showed that the PRF and GTR groups demonstrated significant improvement in the clinical parameters for infrabony defects compared to the access flap-only group, which is consistent with our findings. However, the magnitude of improvements in our study, particularly in Group C (PRF), was more pronounced.

One possible explanation for this divergence could be the differences in PRF protocols. Our study used two additional PRF membranes to cover the defect, providing a more substantial fibrin scaffold for cell proliferation and growth factor delivery. This enhanced use of PRF might have contributed to better soft tissue regeneration and wound healing compared to the single-layer PRF application used in Ustaoglu et al.’s study.

Additionally, Ustaoglu et al. employed a different approach to GTR, using various types of membranes and barrier techniques [24]. These variations in materials and protocols may explain the differences in the overall healing response and periodontal regeneration observed between the two studies. While both studies demonstrate the efficacy of PRF, the more extensive PRF application in our study could explain the superior wound healing and improved glycemic control, likely due to the sustained release of growth factors and the enhanced angiogenic effects provided by the additional PRF layers.

PRF led to the best outcomes in periodontal parameters, healing, and glycemic control due to its rich composition of growth factors and unique fibrin matrix, which plays a critical role in tissue regeneration and systemic healing. PRF contains growth factors such as platelet-derived growth factor (PDGF), transforming growth factor-beta (TGF-β), and vascular endothelial growth factor (VEGF), which are known to promote angiogenesis and enhance the regeneration of soft tissues [25].

These growth factors stimulate endothelial cell proliferation and the formation of new blood vessels, thereby improving blood flow to the healing site and accelerating the repair process [26]. This enhanced angiogenesis not only aids in soft tissue regeneration but also improves oxygenation and nutrient delivery to the wound, which are essential for effective periodontal healing [27].

Moreover, the leukocytes present in PRF release pro-inflammatory cytokines that modulate the inflammatory response, promoting a controlled environment conducive to healing [28]. This anti-inflammatory effect likely reduces systemic inflammation, which could help improve glycemic control. By reducing chronic inflammation, a known contributor to insulin resistance [29], PRF may help enhance the systemic glycemic response, leading to better HbA1c outcomes in patients with diabetes.

The fibrin network in PRF acts as a scaffold for cell migration and differentiation, facilitating the repair of both hard and soft tissues [30]. These molecular and cellular mechanisms collectively contribute to the superior periodontal healing observed in Group C. They may explain the improved glycemic control, as the resolution of periodontal inflammation is known to impact systemic glucose metabolism positively.

Our findings highlight the therapeutic effectiveness and safety of both the PRF and the single-access flap technique in managing periodontal defects. The absence of adverse complications and the favorable progress of postoperative healing are crucial factors for evaluating and comparing the effectiveness of different periodontal treatment methods. These results demonstrate that the technique used in periodontal treatment can significantly influence the healing process and the postoperative recovery of patients. It is essential to consider the benefits and limitations of each technique before deciding on the optimal therapeutic approach for each patient.

Compared to the treatment involving the access flap alone, patients treated with GTR utilizing a resorbable membrane or PRF demonstrated a notable enhancement in their periodontal clinical and radiographic parameters at the six-month follow-up. These findings suggest that the use of PRF as an alternative therapy in the treatment of infrabony defects may be beneficial. They may provide results comparable to the GTR technique, which is considered the gold standard in treating these lesions. GTR techniques are often expensive and require advanced technical expertise, so PRF is more affordable and accessible to implement in clinical practice.

This study employed the standard protocol for preparing PRF using a fixed-angle centrifuge. This method is widely recognized for yielding a matrix rich in platelets and growth factors, essential for promoting the healing and regeneration of damaged periodontal tissue. Adhering to this standardized protocol enabled us to consistently obtain results and evaluate the therapeutic efficacy of PRF objectively and reproducibly. Previous studies have demonstrated its ability to enhance the proliferation and migration of human gingival fibroblasts, human periodontal ligament stem cells, and human bone marrow-derived stem cells in vitro [31].

According to Miron et al. [32], horizontal centrifugation generated both solid and liquid formulations with higher concentrations and counts of platelets and leukocytes compared to fixed-angle centrifuges. They also noted that liquid PRF could act as a carrier for various cell types and nano-sized particles, potentially minimizing immune system responses and foreign body reactions in host tissues following injection.

While this study provides valuable insights into the effects of different periodontal treatments on the clinical parameters and glycated hemoglobin (HbA1c) levels in patients with type II diabetes, several limitations should be acknowledged. This study involved a relatively small sample size (24 patients), which may limit the generalizability of the findings. Larger-scale studies are necessary to confirm the results and ensure broader applicability. The follow-up period was limited to six months. Extending the follow-up period beyond six months would provide more insight into the long-term efficacy of these treatments. Future studies could assess the sustainability of improvements in both periodontal parameters and glycemic control over 12 or 24 months.

This study focused on the clinical parameters and did not include radiographic assessments, which could have provided additional insights into bone regeneration and defect resolution. This study did not include microbiological evaluations or molecular analysis of the inflammatory markers, which could have provided more profound insights into the biological mechanisms behind the observed clinical outcomes. Assessing bacterial profiles and molecular markers such as cytokines or matrix metalloproteinases (MMPs) would help better understand the treatments’ impact on periodontal disease progression and systemic inflammation.

For a better understanding of the most effective treatment for periodontitis-induced infrabony defects in patients with type II diabetes, it is recommended to conduct future clinical trials on platelet-rich fibrin (A-PRF), leukocyte- and platelet-rich fibrin (L-PRF), and injectable platelet-rich fibrin (I-PRF).

Moreover, this technique can be combined with specific adjunctive periodontal therapies such as laser, photoactivation (during the etiological phase), and application of local bioactive substances for an improved clinical outcome [33,34,35,36]. This can be especially useful in systemically impaired individuals, such as those with diabetes mellitus, autoimmune diseases, and cardiovascular, renal, and other pathology patients. Moreover, these techniques can be applied to patients undergoing implant therapy, and future studies can be led to explore this area [37].

Further studies could investigate the systemic effects of PRF, particularly its impact on glycemic control. Exploring the potential mechanisms by which PRF contributes to reductions in HbA1c could open new avenues for treating systemic conditions through localized periodontal therapy. Furthermore, conducting cost-effectiveness analyses would be valuable for determining the practical implications of using PRF and GTR in routine clinical practice. This could help assess widespread use and its financial feasibility, especially in resource-limited settings. By pursuing these directions, future research could build on the promising results of our current study and further enhance the understanding of optimal periodontal treatment approaches for patients with diabetes.

## 5. Conclusions

In conclusion, this study demonstrated that the addition of a resorbable membrane and platelet-rich fibrin to open-flap debridement improved the periodontal clinical parameters in patients with type II diabetes. Furthermore, these treatments also contributed to better glycemic control, as indicated by reductions in HbA1c levels. These results suggest that using advanced regenerative techniques, particularly PRF, may offer additional benefits in managing both periodontal disease and systemic health in diabetic patients.

## Figures and Tables

**Table 1:** Demographic results.

Parameter	Study Population (*n* = 24)	
Age [Mean ± SD (CI)] (years)	45 ± 1.35 (44.46, 45.54)	
Gender [*n*, (%)]	Male	13 (54.17%)
	Female	11 (45.83%)
Environment [*n*, (%)]	Urban	17 (70.83%)
	Rural	7 (29.17%)

SD: Standard deviation; CI: 95% confidence interval.

**Table 2:** Comparisons of clinical parameters between groups at baseline, 3 months, and 6 months.

Parameter	Assessment Time	Group A	Group B	Group C	*p*-Value
PI	T0	0.82 ± 0.23 (0.73, 0.91)	0.78 ± 0.19 (0.70, 0.86)	0.77 ± 0.17 (0.70, 0.84)	0.532
	T1	0.73 ± 0.27 (0.62, 0.84)	0.69 ± 0.18 (0.62, 0.76)	0.68 ± 0.19 (0.60, 0.76)	0.579
	T2	0.70 ± 0.21 (0.62, 0.78)	0.53 ± 0.27 (0.42, 0.64)	0.55 ± 0.30 (0.43, 0.67)	0.028
BOP	T0	4.49 ± 0.61 (4.25, 4.73)	4.42 ± 0.65 (4.16, 4.68)	4.49 ± 0.71 (4.21, 4.77)	0.814
	T1	3.29 ± 0.87 (2.94, 3.64)	3.02 ± 0.95 (2.64, 3.40)	2.95 ± 0.78 (2.64, 3.26)	0.363
	T2	2.95 ± 0.79 (2.63, 3.27)	2.32 ± 0.69 (2.04, 2.60)	2.32 ± 0.58 (2.09, 2.55)	0.001
PD	T0	7.42 ± 0.63 (7.17, 7.67)	7.62 ± 0.57 (7.39, 7.85)	7.59 ± 0.47 (7.40, 7.78)	0.552
	T1	5.54 ± 0.53 (5.33, 5.75)	5.32 ± 0.71 (5.04, 5.60)	5.22 ± 0.38 (5.07, 5.37)	0.019
	T2	4.85 ± 0.81 (4.53, 5.17)	4.39 ± 0.55 (4.17, 4.61)	4.29 ± 0.62 (4.04, 4.54)	0.005
CAL	T0	8.12 ± 0.83 (7.79, 8.45)	8.19 ± 0.24 (8.09, 8.29)	8.25 ± 0.37 (8.10, 8.40)	0.831
	T1	6.52 ± 0.46 (6.34, 6.70)	6.19 ± 0.56 (5.97, 6.41)	6.02 ± 0.73 (5.73, 6.31)	0.063
	T2	5.89 ± 0.57 (5.66, 6.12)	4.99 ± 0.31 (4.87, 5.11)	4.92 ± 0.35 (4.78, 5.06)	<0.001

Data are presented as mean ± standard deviation (confidence intervals for 95% confidence interval); PI: plaque index; BOP: bleeding on probing; PD: probing depth; CAL: clinical periodontal attachment level; T0: baseline assessment; T1: evaluation 3 months postoperatively; T2: evaluation 6 months postoperatively. Statistical significance set at *p* < 0.05.

**Table 3:** Intra-group changes in clinical parameters at 3 and 6 months postoperatively compared to baseline.

Parameter	Assessment Time	Group A		Group B		Group C	
		Mean Difference	*p*-Value	Mean Difference	*p*-Value	Mean Difference	*p*-Value
PI	T1	0.08 ± 0.20(−0.00002, 0.16)	0.221	0.08 ± 0.21 (−0.004, 0.16)	0.041	0.08 ± 0.24 (−0.016, 0.18)	0.055
	T2	0.09 ± 0.15 (0.03, 0.15)	0.001	0.23 ± 0.18 (0.16, 0.30)	<0.001	0.19 ± 0.17 (0.12, 0.26)	<0.001
BOP	T1	1.18 ± 0.97 (0.79, 1.57)	<0.001	1.38 ± 0.87 (1.03, 1.73)	<0.001	1.51 ± 0.64 (1.25, 1.77)	<0.001
	T2	1.51 ± 1.03 (1.10, 1.92)	<0.001	2.07 ± 0.81 (1.75, 2.39)	<0.001	2.14 ± 0.75 (1.84, 2.44)	<0.001
PD	T1	1.85 ± 0.56 (1.63, 2.07)	<0.001	2.28 ± 0.51 (2.08, 2.48)	<0.001	2.35 ± 0.61 (2.11, 2.59)	<0.001
	T2	2.55 ± 1.21 (2.07, 3.03)	<0.001	3.21 ± 0.76 (2.91, 3.51)	<0.001	3.28 ± 0.77 (2.97, 3.59)	<0.001
CAL	T1	1.58 ± 0.79 (1.26, 1.90)	<0.001	1.97 ± 0.87 (1.62, 2.32)	<0.001	2.21 ± 0.77 (1.90, 2.52)	<0.001
	T2	2.21 ± 1.17 (1.74, 2.68)	<0.001	3.18 ± 0.70 (2.90, 3.46)	<0.001	3.31 ± 0.59 (3.07, 3.55)	<0.001

Data are presented as mean ± standard deviation (confidence intervals for 95% confidence interval); PI: plaque index; BOP: bleeding on probing; PD: probing depth; CAL: clinical periodontal attachment level; T1: evaluation 3 months postoperatively; T2: evaluation 6 months postoperatively. Statistical significance set at *p* < 0.05.

**Table 4:** Inter-group and intra-group comparisons of HbA1c values for the three evaluation moments.

	Assessment Time	Group A	Group B	Group C	*p*-Value
**HbA1c**	T0	7.51 ± 0.81 (7.19, 7.83)	7.50 ± 0.68 (7.23, 7.77)	7.53 ± 0.71 (7.25, 7.81)	0.843
	T1	7.33 ± 0.75 (7.03, 7.63)	7.21 ± 0.65 (6.95, 7.47)	7.10 ^a^ ± 0.66 (6.84, 7.36)	0.035
	T2	7.22 ^b^ ± 0.70 (6.94, 7.50)	7.10 ^b^ ± 0.67 (6.83, 7.37)	6.44 ^b^ ± 0.56 (6.22, 6.66)	<0.001

Data are presented as mean ± standard deviation (confidence intervals for 95% confidence interval); HbA1c: glycated hemoglobin (measured as a percentage); T0: baseline assessment; T1: evaluation 3 months postoperatively; T2: evaluation 6 months postoperatively; ^a^: *p* < 0.05 intra-group versus baseline; ^b^: *p* < 0.05 intra-group versus baseline. Statistical significance set at *p* < 0.05.

## Data Availability

The data used to support the findings of this study are available from the corresponding author upon reasonable request.
